# Workforce Perceptions of a Conceptual Hospital-at-Home Digital Twin: Qualitative Study

**DOI:** 10.2196/96645

**Published:** 2026-11-05

**Authors:** Faiza Yahya, Matthew Cooper, Lauren Lawson, Hamde Nazar

**Affiliations:** 1 National Institute for Health and Care Research (NIHR) Newcastle Patient Safety Research Collaboration, Newcastle University, Newcastle upon Tyne, NE1 7RU, United Kingdom, +441912086000; 2 School of Psychology, Newcastle University, Newcastle upon Tyne, United Kingdom; 3 School of Pharmacy and Biomolecular Sciences, Royal College of Surgeons in Ireland (RCSI), Dublin, Ireland

**Keywords:** hospital-at-home, digital twin, artificial intelligence, machine learning, risk prediction, digital health, staff, workforce, implementation, adoption

## Abstract

**Background:**

Hospital-at-home (HaH) services are transformative for delivering acute care in a patient’s home, with support from a multidisciplinary team and remote monitoring technologies, and there is a growing demand for innovation and digital solutions. Digital twin (DT) technology offers the possibility of leveraging real-time data to personalize care, predict risk, and improve patient management. However, perceptions of the workforce regarding the application of HaH DTs remain underexplored, and addressing this is crucial for successful adoption and sustainability.

**Objective:**

This qualitative study aimed to explore health and care professionals’ perceptions of the concept of a HaH DT model of care and its perceived impact.

**Methods:**

A total of 26 semistructured interviews were conducted between June and December 2025. Participants were based in the United Kingdom and were recruited through purposive sampling via known contacts, professional networking, and social media. A short vignette on DTs was presented to participants at the start of the interview. An interview topic guide centered around acceptability and the domains of the Non-adoption, Abandonment, Scale-up, Spread, and Sustainability (NASSS) framework was used to explore complexity in implementation and potential management. Data analysis involved a hybrid approach drawing on reflexive thematic analysis.

**Results:**

Seven overarching themes were identified, highlighting a mixture of perceived benefits, concerns, and recommendations. Participants noted value in personalized, holistic care enabled by real-time data-driven interventions and appropriate patient-clinician contact. However, concerns included trust in DTs, with participants highlighting that implementation requires adapting workforce roles, strong education and training, system-level infrastructure, and a supporting evidence base.

**Conclusions:**

This work provides the foundations for stakeholders and developers to better understand the likely adoption of a HaH DT and the likely impact on individuals, organizations, and systems. Findings from this study highlight key recommendations, and further research and implementation efforts should prioritize stakeholder involvement to develop clear actionable plans.

## Introduction

Hospital-at-home (HaH) services (sometimes referred to as virtual wards) are innovative models aimed at delivering hospital-level care within a patient’s home, using a multidisciplinary team approach and remote monitoring technology [1]. Evidence has shown that patients prefer to be cared for at home, and HaH can reduce the need for residential care after patients are discharged from the hospital [2]. There is a significant need to ensure patients are cared for safely and effectively, in the right place, and at the right time. However, caring for patients at home who are acutely unwell comes with its own clinical and operational challenges, particularly for aging populations with increasingly complex health needs [3]. Despite the potential benefits of HaH models, their implementation has been variable, especially the adoption of technology and the consistency in how patients are managed [1,4-9]. Unlike hospital wards, these innovative workflows mean that clinicians are often managing patients remotely with limited direct observation, relying on decisions that are sometimes made on patient-reported intermittent physiological measurements. This can create safety blind spots, making it difficult to identify early deterioration, prioritize caseload, and maintain oversight. Increasing service demands and workforce pressures further challenge clinicians’ abilities to maintain remote situational awareness, requiring approaches that can support and streamline their cognitive workload and case management for delivering timely interventions. Compared with typical clinical settings, there is a greater need for proactive and effective risk assessment, status prediction, and care management rather than reactive care [10].

In the era of advancing digital solutions, there has been a strong emphasis on embracing technology to support streamlined, more efficient health care delivery. In England, the National Health Service (NHS) 10-year health plan focuses on 3 major shifts: hospital to community, sickness to prevention (more proactive care), and analog to digital [11]. Current HaH models use remote patient monitoring (RPM) technologies, and some have virtual dashboards to support decision-making. However, their adoption has been variable. These models primarily present current or historical data, failing to reflect a patient’s live status, which can fluctuate rapidly in acute care. In contrast, a digital twin (DT) is a dynamic virtual representation of a person and/or their environment, and it continuously integrates real-time data to forecast future possibilities and inform patient management decisions [10,12,13]. Rather than just displaying static physiological observations, a DT can simulate how a patient’s condition may evolve over time under different scenarios, enabling the prediction of risk, assessment of intervention options, and prioritization of workflows [10,12,13]. Consequently, DTs have the capability to personalize care for an individual patient and/or setting and offer bidirectional data exchange, predictive simulation, and autonomous decision support functions to support patient management. These capabilities extend above and beyond conventional RPM and dashboards as they are supported by data-driven care planning, leveraging AI and machine learning algorithms. The DT concept involves 5 core architectural components and is presented in Figure 1 [10,13]. The risk prediction capability supports clinicians and service providers in introducing early interventions, leading to improved safety. However, beyond this, DTs can optimize care pathways by integrating patient physiological parameters, home environmental parameters, and service-level operational information. Current evidence points to an expanding body of research [14-16], the development of risk prediction models [17,18], and an increasing application of command centers [10,19] to manage caseloads. Despite this, there is little evidence on the use of DT technology in health care in real-world practice, with many DT applications in health care being in early stages of development and nonexistent in HaH models of care [20]. Building on previous research, we know that the adoption of technology or innovation comes with its own challenges, especially when it comes to human factors and multilevel factors (eg, acceptability, cognitive workload, and human-automation interaction) [21]. As these factors influence sustainability, a fundamental understanding of workforce perspectives is essential, taking into consideration the inherent sociotechnical complexity and the interactions among people, services, and technology. Guided by the Non-adoption, Abandonment, Scale-up, Spread, and Sustainability (NASSS) framework of health and care technologies [21], the aim of this study was to explore the perceptions of health and care professionals and the perceived impact of the concept of a DT-enabled HaH model of care in order to anticipate and plan for the potential adoption or nonadoption of such technology in the future.

**Figure 1.**
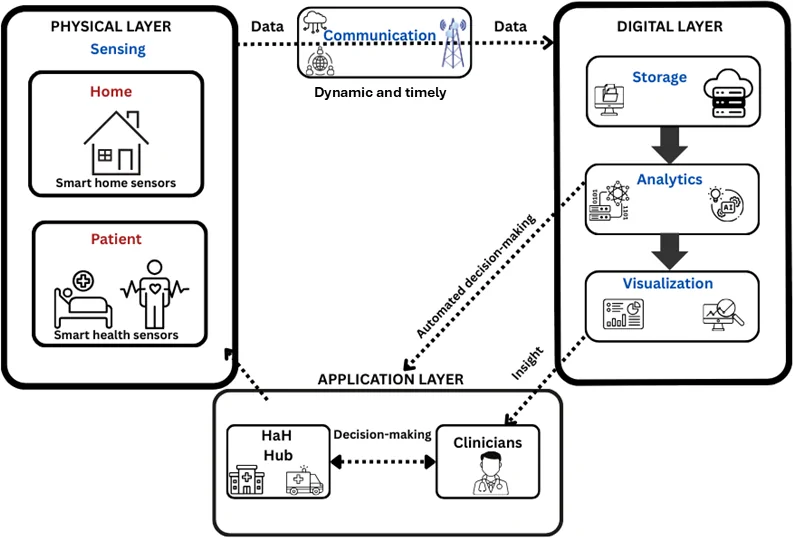
Simplified conceptual model for the hospital-at-home (HaH) digital twin architecture [10].

## Methods

### Study Approach

This qualitative study used semistructured interviews and reflexive thematic analysis. The study has been reported in line with the COREQ (Consolidated Criteria for Reporting Qualitative Research) checklist (Checklist 1) [22].

### Study Design

#### Theoretical Framework

This study sought to gain an understanding of health and care professionals’ perspectives on a HaH DT concept and its acceptability. The semistructured interviews were guided by a combination of the theoretical framework of acceptability (TFA) [23] and the NASSS framework [21]. The TFA [23] is useful to explore the individual-level factors of those delivering or receiving a health care intervention, and the NASSS framework [21] is useful to explore organizational factors and the wider system across the following 7 domains: condition, technology, value proposition, adopters, organization, wider system, and adaptation over time (Multimedia Appendix 1).

The NASSS framework was selected as it provides a sociotechnical perspective on the complexity of the implementation of health and care technologies and has been widely applied to digital health interventions, including telemedicine and virtual care [21,24].

Underpinned by theories of individual, organizational, and system-level change [21,24,25], the NASSS framework provides a structured approach to understanding implementation complexities and potential pragmatic solutions in complex (adaptive) systems such as the HaH DT concept [24,25]. This approach enabled us to gain a deeper understanding of the perspectives of the workforce at the micro, meso, and macro levels, using a robust methodology and reflexive thematic analysis.

### Participant Selection

Participants were recruited by purposive sampling via known contacts, professional networks, forums, conferences, and snowball sampling. Social media were used to support recruitment via professional and social platforms (eg, LinkedIn). Recruitment via these methods aimed to gain a variety of perspectives from a diverse group of health and care professionals based in the United Kingdom and from different backgrounds in any setting (hospital, primary care/community, or HaH).

Consistent with reflexive thematic analysis, decisions regarding sample size were guided by the adequacy and richness of the dataset. Data collection and analysis occurred concurrently, with familiarization and preliminary coding undertaken alongside ongoing recruitment. This was determined by the lead researcher (FY) and regular discussion among the co-researchers after every third interview. Recruitment ceased when additional interviews were no longer contributing sufficiently novel insights relevant to the study aims, and this was determined by the lead researcher’s interpretative judgment aligning with reflexive thematic analysis [26].

### Data Collection

Interviews took place online via Microsoft Teams. An interview guide, which was informed by the TFA [23] and NASSS framework [21], enabled addressing the key themes in the research aims and objectives. Moreover, a short vignette on DTs was presented to participants at the start of the interview (Multimedia Appendix 2). This included a short 4-minute video prepared by the lead author, which explained the concept of HaH, the potential of digital technology, and the advancements of DTs in health care, leaving participants with an understanding of how a DT can operate, its goals, and its constituent parts. Further information can be found in Multimedia Appendix 2. Demographic data were recorded to measure the diversity and inclusion of the sample. The data included professional background, role, geographical location, and level of expertise/training. Interview data were recorded and transcribed verbatim via Microsoft Teams. The interview transcripts were not returned to the participants for comment or checking.

### Data Analysis

#### Overview

The anonymized data were managed in NVivo software (Lumivero), and the development of codes, categories, and themes was an iterative process. Data analysis drew on reflexive thematic analysis following the 6 steps outlined by the Braun and Clarke approach as a general guide [27,28]. The steps are outlined below and illustrated in Figure 2. The process was iterative, moving back and forth between the data and phases to facilitate rigor in data interrogation and engagement [28]. The process was adapted to incorporate the NASSS framework as an interpretative lens to contextualize the findings and build an in-depth understanding for real-world application.

**Figure 2.**
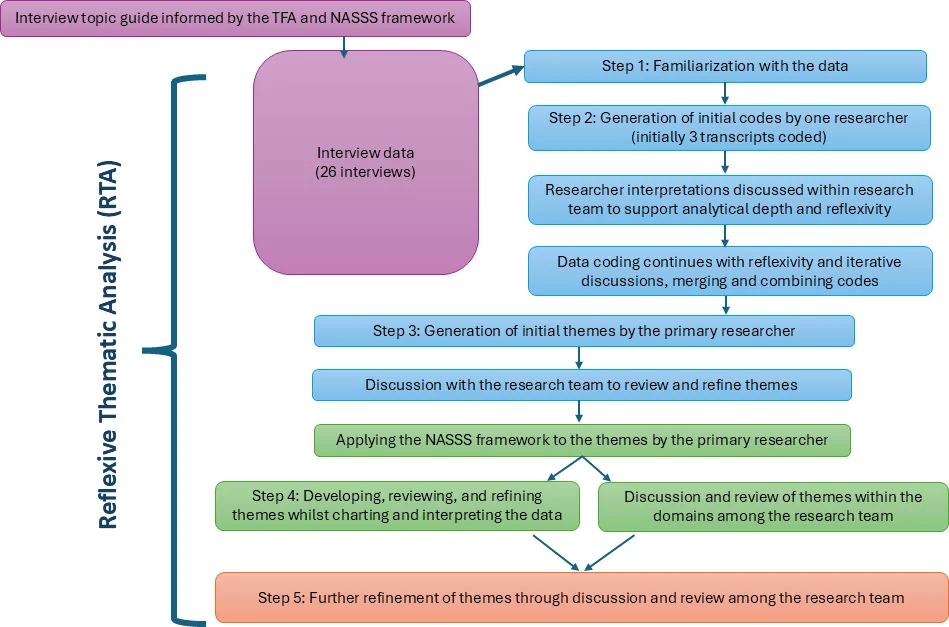
Stepwise methods used for qualitative data analysis. NASSS: Non-adoption, Abandonment, Scale-up, Spread, and Sustainability; TFA: theoretical framework of acceptability.

#### Step 1

The first step involved familiarization with the data in the transcripts through a systematic review of each transcript. The primary researcher (FY) read and reread each transcript and made reflective notes after reviewing each transcript, becoming immersed and engaged with the data.

#### Step 2

Initial codes were generated by the primary researcher (FY), with consideration of the research aims and objectives. An inductive coding approach was used, with both semantic and latent coding where applicable. This was done reflexively, with interpretations discussed within the research team to support analytical depth and reflexive engagement with the data. These discussions were intended to enrich interpretation rather than achieve coding agreement or consensus, and they enabled merging and refining of codes before moving to the next step. Examples are provided in Multimedia Appendix 3.

#### Step 3

Initial themes were generated by the primary researcher (FY), which involved critically engaging with the raw data to organize the initial codes into themes. This was then reviewed multiple times to ensure they captured meaningful patterns before applying the 7 domains of the NASSS framework to the generated themes. Examples are provided in Multimedia Appendix 4. The iterative analytical process using the NASSS framework aimed to allow greater interpretation by exploring use in the wider context, organizational readiness, and long-term viability [21]. This was done to enable key recommendations to be drawn for further work or implementation in practice.

#### Step 4

The development, review, and refinement of themes involved discussion with the research team (FY, MC, HN, and LL) as a form of sense-checking to clarify interpretations and challenge assumptions. The coded data extracts were reviewed to refine, combine, or discard any themes as appropriate, ensuring they were meaningful and representative of the data.

#### Steps 5 and 6

The refinement, definition, and naming of themes involved further iterative, detailed analysis of each theme and drafting of the analytical narrative to define the scope of each theme within the NASSS domains and contextualize the data in relation to the coded data extracts. This process involved flexibility and iterative discussions with the research team. Multimedia Appendices 5 and 6 illustrate parts of this process and provide detailed descriptions.

### Research Team and Reflexivity

It is acknowledged that researcher reflexivity inevitably shapes the research and engagement with data [27]. The primary researcher (FY), a female PhD candidate, has a health care professional background (clinical pharmacist) with experience in various health care sectors but has very minimal experience in providing care at a patient’s home and has no clinical experience in HaH care. This experience can be viewed as an asset [27], as the researcher presents a degree of subjectivity that allows engagement with participants, supporting the attainment of richness and meaning from the data. The researcher’s values as a health care professional and previous knowledge of what matters to patients and the workforce were also considered. Functional reflexivity and previous interviews with HaH clinicians also supported and shaped the development of this research to further enhance understanding. Reflexivity was considered throughout the study, and a reflexive journal was kept by the primary researcher (FY) after each interview to note any influences of their own existing beliefs or experiences in order to minimize any bias in data interpretation. To acknowledge this reflexivity while enhancing transparency and analytical rigor, iterative discussions with experienced qualitative researchers (MC, LL, and HN) were undertaken. Ongoing discussions took place during inductive coding and analysis to support answering the research questions in relation to the NASSS framework.

### Ethical Considerations

This study obtained ethical approval from The Faculty of Medical Sciences Research Ethics Committee at Newcastle University (reference number: 61011/2023).

Informed consent was collected from participants before the interview and was rechecked at the interview. Privacy and confidentiality were maintained by anonymizing any participant-identifiable data. To protect anonymity, generic group codes were assigned to professional roles and applied to transcripts (eg, manager, clinical lead, staff nurse, and pharmacist). Any participant details were kept secure on a university password-protected domain in line with university regulations. Participants were remunerated for their participation in the form of a gift voucher (£27.50 [US $36.30]) funded by the National Institute for Health and Care Research (NIHR) Newcastle Patient Safety Research Collaboration in line with the national NIHR guidance [29].

## Results

### Overview of the Participant Sample

Between June and December 2025, 44 individuals expressed an interest in the study. Twenty-eight consented to participate, but a final total of 26 health and care professionals participated in the semistructured interviews. Participant characteristics are presented in Table 1. Among the participants, 4 had leadership responsibilities within their team, organization, or integrated care board (ICB); 2 were clinical directors; and 1 was a geriatrician.

**Table 1.** Participant characteristics.

Characteristic		Value (N=26), n (%)
Profession		
Nurse		8 (31)
Pharmacist		6 (23)
Care coordinator		2 (8)
Physiotherapist		2 (8)
Consultant		2 (8)
Medical registrar		1 (4)
Occupational therapist		1 (4)
Paramedic		1 (4)
Pharmacy technician		1 (4)
Social prescriber		1 (4)
General practitioner		1 (4)
Sector		
Secondary care		8 (31)
Primary care		5 (19)
Community		6 (23)
Both/integrated care		5 (19)
Integrated care board		2 (8)
Region (based on integrated care board locality) [30]		
North East and Yorkshire		6 (23)
North West		6 (23)
Midlands		11 (42)
London		1 (4)
South East		1 (4)
South West		1 (4)

Interviews lasted between 45 and 65 minutes. The average number of years of experience in health care overall was 16.7 years (median 15 years). Among the 26 participants, 16 (62%) had experience in HaH (average experience of 2.5 years; range: 0.5‐10 years), and of these, the majority (13/16, 81%) had 5 years or less of experience in HaH/virtual wards.

### Key Themes From Thematic Analysis

Seven overarching themes were generated from in-depth analysis of the interview data. These are presented in Figure 3 and described in further detail below. Themes 1 and 2 relate to the positive benefits of DTs as perceived by the workforce (this covers factors such as remote situational awareness and individualized predictive algorithms), and themes 2 and 3 relate to current concerns that are likely to change with time (this encompasses factors such as human-automation interaction and sociotechnical factors). It is important to note that there were mixed views (both positive and negative in theme 2). Finally, themes 4‐7 relate to what the workforce believes needs to happen to support the adoptability of HaH DTs (these may relate to implementation, sociotechnical complexity, and supporting information processing).

**Figure 3.**
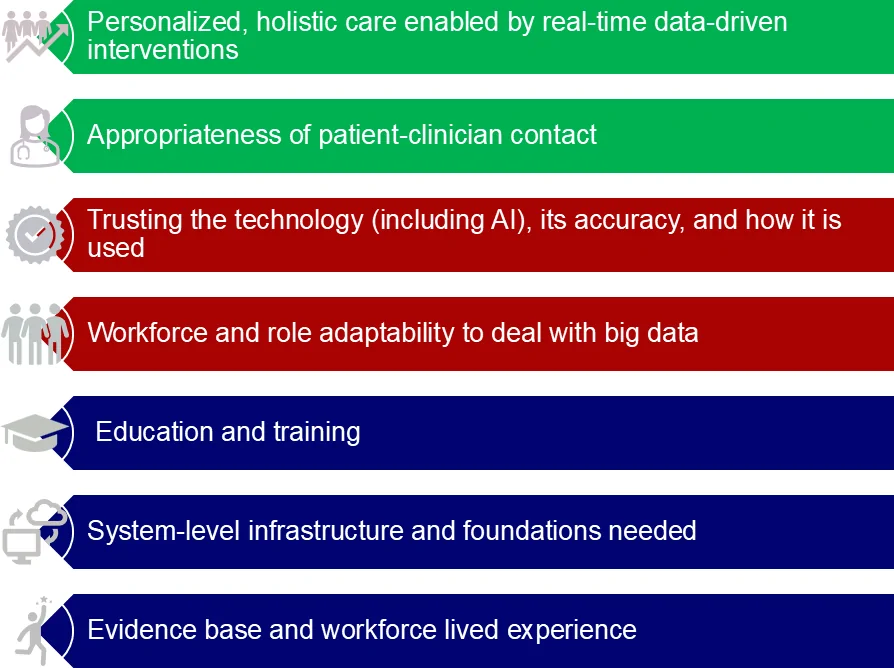
Key themes.

### Theme 1: Personalized, Holistic Care Enabled by Real-Time Data-Driven Interventions

Participants recognized that DTs enable personalized, individualized care, enhancing a holistic picture. DTs were seen to have substantial transformational value to patients, allowing personalization of care within a patient’s context and improving access to care for those who may not normally engage with hospitals when needed. This real-time and connected data facilitated “getting patients the right care at the right time” (participant #10), and the data could potentially “connect the dots” between multiple conditions or health care services in a disjointed health care system. This perceived potential of DTs goes beyond conventional monitoring, giving the opportunity to detect emerging functional or social care needs.

The real-time data capture supports clinicians in tracking micro-level trends (for the patient) and predicting trajectories, and it also provides strategic insights at the meso-level (for workforce and organizational planning) and macro-level (for better population health management).

*Being able to use a tool like this and figure out on our current caseload which of the people who we could safely defer for a visit or safely not need someone to go out to today in order to take more people in. Now that you know, so not just the in the moment prediction of escalation, but looking at a patient or looking at a person over time on an NHS at home pathway and trying to predict and work out how long they might need to stay, when might they be discharged? When not when? So there’s something about that forecasting not just an acute deterioration, but forecasting, Yeah, length of stay and the pattern of patterns beyond … just a deterioration.*

[Participant #26]

### Theme 2: Appropriateness of Patient-Clinician Contact

This theme grouped both positive and negative feelings from participants. Although there was a perception of reducing physical patient-clinician contact, which can sometimes impact relationships and increase anxiety, it was appreciated that a DT can increase the appropriateness and value of subsequent in-person contact by providing teams with valuable information to send the right clinician to the right patient at the right time. Thus, increasing the value of patient-clinician contact.

*That’s two visits that have had to happen, whereas we could have avoided that by sending the right person the first time to go and review them.*

[Participant #9]

Participants reported the need to ensure “the right patients and the right problems” (participant #25) are considered when implementing DTs. Considering this, participants felt that patients with multimorbidity, cognitive and learning disabilities, or severe mental health problems would have complexities deemed to be unpredictable and high risk for a DT. Participants felt that DTs would be better accepted if patients were given support or an informed choice, drawing comparisons to previous experiences with digital transformation in practice.

*...just don’t tell people, just don’t send out a message saying you can’t access your prescriptions by phoning this … anymore. Explain why, explain how you can help them.*

[Participant #23]

### Theme 3: Trusting the Technology (Including AI), Its Accuracy, and How It Is Used

A recurrent theme from participants was human-automation trust and trust in the accuracy of the technology, emphasizing that it takes time to build trust with new technologies like DTs. To clinicians, DTs were seen as a revolutionary decision aid to be used as a tool to support decision-making, but they emphasized that DTs should not replace it. Participants reported that “this is a tool to help with your decision-making, but it is the whole clinical picture that [you need]” (participant #9) and repeatedly mentioned the importance of “clinician instinct and gut-like pattern recognition” (participant #18). They felt technology could not always replicate this, emphasizing that there remains space for skills with clinical judgment and patient choice.

There were differing opinions regarding workforce acceptability, highlighting that this can vary depending on confidence, trust, experience level, digital literacy, and readiness. These initial hesitancies revolved around confidence in trusting the technology and clinical accountability.

*…they need to be reliant that they’re keeping their patients safe because that you do often get a fear that well, if anything were to happen. If that device didn’t pick something up, I as a nurse or a registered practitioner would then be liable accountable for that patient deteriorating because I’ve signed off to use a device and it’s failed.*

[Participant #21]

Participants felt that with time, embedded training, and increasing familiarity with DTs and AI, the current younger generations and upcoming generations will be more accepting and trusting of DTs. Similarly, participants perceived that this would be the case with patient adopters.

### Theme 4: Workforce and Role Remodeling to Deal With Big Data

Participants expressed concerns about the amount of data coming into DTs, worrying that this could “just open the floodgates” (participant #14)*,* with consequences for capacity with increasing data volumes.

*...you’re kind of opening yourself up to getting tons and tons of data, that potentially isn’t all relevant, but also that we don’t have the staff to deal with either.*

[Participant #25]

This requires different ways of working, with staff having to adopt different roles and be adaptable in order to enable data to be reviewed and information to be processed and actioned where necessary. To do this effectively and efficiently, a dedicated role and 24-hour resource were viewed as crucial. Some participants saw this as an opportunity for “a better use of resources and more and more timely” (participant #12), while others had concerns that this is a change from their usual work and clinical training (requiring behavioral adaptation).

*There’s going to be a lot more sitting down and looking at a computer screen rather than going and seeing patients, and I think that’s for a lot of people. That’s not why they came into this profession.*

[Participant #25]

### Theme 5: Education and Training

An underlying theme throughout discussions with participants was the need for robust and continual training with DT technology.

*...ongoing support that we need when we’re introducing new things and change… And a proper… ongoing plan and that that is visible to staff and that they know that they’re not going to be left in the lurch.*

[Participant #26]

A lack of training and understanding was a key factor influencing some of the hesitancies that participants experienced, negatively impacting adoption. Participants explained that education and training need to clearly present the benefits of DTs and be provided to patients, staff, and organizations. This should factor in dedicated resources for training and protocol updates, as well as a dedicated contact for dealing with technical issues. The presence of trained “DT champions” was suggested to support adoptability and reduce training burden on organizations and teams. The data highlighted that the different ways of working with DTs require future training to be embedded at the undergraduate and preregistration levels to ensure that incoming clinicians are accustomed to using such technology, ultimately enhancing its sustainability.

### Theme 6: System-Level Infrastructure and Foundations Needed

Current systems still require improvements to ensure digital readiness and capability to leverage the potential of DTs. Participants felt strongly about the lack of interoperability and organizational ergonomics that exist in current health care systems and felt that there is an overwhelming need to ensure that the foundations are rooted first.

*You see, the foundations are just so different everywhere. And they’re so unsteady in areas that you wouldn’t think that you’d have to reinforce in order for all of this to work. It’s like you’ve got a drip, a tap dripping at your sink. And before you know it, you’re out in the streets digging up the pipes you know because the source of the problem is actually so far away, but you can’t progress until you fix that first.*

[Participant #15]

This includes interoperability of systems and data sharing to ensure seamless flow of real-time data and shared visibility centered around the patient. Moreover, reliable Wi-Fi access and connectivity are paramount for DTs to be adoptable. This concern focused on avoiding a digital and social divide, which was previously experienced with technology by some participants.

*…they were just incredibly rural, so they had no mobile or Internet connection. They couldn’t use their services. So straight away it disadvantaged those groups of people.*

[Participant #2]

This commitment to system-level infrastructure is required at organizational and wider system levels. In addition to the digital infrastructure required, participants highlighted the need for human resources, flexible workforce structures, and adaptability to support digital readiness and its continued capability.

*...what is the infrastructure that’s going to be in place to what infrastructure do you need to support that? And I don’t mean, not only just data infrastructure, but what’s the human infrastructure for it, because I think if you’re adding in all these additional layers, you’re going to need additional IT support additional people. The key bit there was you’re sending them home with the enough support and the right care, and then that very quickly.*

[Participant #2]

The value proposition was perceived to be high for both the system and individual patients. From a system perspective, participants mentioned that while the initial investment is significant, there is a substantial return on the investment in the long term—giving rise to better population health management, improved workflows, reduced hospital admissions, and better patient outcomes, ultimately supporting the statement, “you’ve got to spend money to save money” (participant #3). However, having a clear “contingency plan as well for when it’s not working” (participant #20) was seen as important, which would require integrated care systems to work together.

### Theme 7: Evidence Base and Workforce Lived Experience

DTs are still an unfamiliar concept. To aid successful adoption and sustainability, participants suggested that DTs would require a promising evidence base. This could include positive case studies of use or lived experiences of the workforce, which can build familiarity and trust over time.

*I think anything, anything that’s new is always taken up with skepticism, skepticism. It’s like not easily implemented, but once it’s implemented and people see the effect and the positive impact. Of course they accept it.*

[Participant #13]

Participants generally expressed a positive outlook, agreeing that DTs align with the 10-year plan and long-term health care strategies. They emphasized the need to embrace change, recognizing that workforce culture will change with time, knowledge, and adaptability.

Participants viewed the alignment of DTs with national health policy as a positive facilitator for implementation. Moreover, participants felt that as familiarity with DT technology improves and its benefits for holistic, personalized care become clear, the mindset and adoption will likely change over time.

*I think it I think it’s happening whether we like it or not to be honest and I think we need to just adapt to this happening because it would be madness not to do it really. It’s part of the NHS strategy.*

[Participant #25]

There was a consensus that evidence (or proof of concept) is needed for clinicians to build trust with DTs. Some participants suggested that initially a phased approach is needed to help build the evidence and proof of concept in the early stages.

This would help identify any unforeseen challenges, uncover technical considerations, and increase familiarity, with participants suggesting “you can double run it, can’t you, to start with” (participant #12). Participants questioned whether an acute setting was the best starting point, and they suggested starting with long-term conditions or “starting with relatively low risk standardized care pathways” (participant #25). Disease groups with “clear sort of clinical scenarios” (participant #25) were preferred for proof of concept—such as respiratory/chronic obstructive pulmonary disease, heart failure, influenza, and pneumonia—to identify key learnings that support wider implementation and scale-up over time. Participants provided suggestions for different settings for the initial implementation (Table 2).

**Table 2.** Quotes from participants regarding different settings to begin the implementation plan.

Setting	Quote from participant	
Predischarge care (step-down hospital at home)	*“…*I think, you know, a day or two days before they leave hospital just to get them accustomed to using the technology.” (participant #21)	
Care homes	*“*If you were to go to a trial, trial it in like a care home setting, because then obviously you have got people that are able to deal with technology and able to follow instructions to try and see how that’s going to pan out.*”* (participant #4)	
Long-term condition monitoring (admission avoidance)	*“*I think you’d probably do it in long term because you would also then learn the acute stuff because you’d start to learn about when people have deteriorating and you’d get that information about what you know, what are the parameters for that person that are changing, how do we pick them up earlier... You’ll have those kind of baseline…findings as well.” (participant #26)	

### Integrating the NASSS Framework in Thematic Analysis

We provide an overview of the 7 domains of the NASSS framework for implementation (Figure 4) and any perceived complexity. These are also described in more detail in Multimedia Appendix 6.

**Figure 4.**
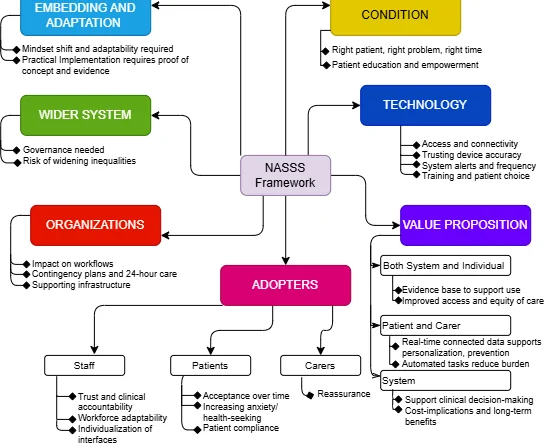
Analytical themes integrated into the Non-adoption, Abandonment, Scale-up, Spread, and Sustainability (NASSS) framework for implementation.

### Using the NASSS Framework for Implementation and Sustainability

From the synthesis of the data, an in-depth understanding was obtained to identify areas of uncertainty and interdependencies. The 7 NASSS domains can be classified as simple (straightforward, predictable, and few components), complicated (multiple interacting components or issues), or complex (dynamic, unpredictable, and not easily disaggregated into constituent components) [25]. This supports the development of potential actionable solutions that stakeholders may wish to consider to mitigate/reduce complexity during the implementation and sustainability of HaH DTs (Table 3).

**Table 3.** Use of the NASSS^a^ framework and participant-informed implementation and sustainability considerations for HaH^b^ DTs^c^.

NASSS domain	Complexity level	Rationale	Proposed action plans	Actors
Condition	Complex	Conditions in acute HaH may present as unpredictable and high risk, with complex multimorbidities. Complexity is further enhanced by continuous monitoring of patient data or by the presence of cognitive impairments.	Selection criteria for suitability Appropriate training and education Escalation pathways Tailoring monitoring to individual patient need (eg, timed at 15 min intervals) and using patient baseline data	Practitioners, clinicians, service leads, and clinical leads
Technology	Complex	Currently, the technology is still unfamiliar and not yet fully developed. Existing technologies, such as wearables, may not have a clear linkage between data and current health care systems. The predictive capabilities remain conceptual and innovative, presenting uncertainty. Over time, supportive infrastructure is likely to adapt with improved understanding, evidence, and familiarity in practice.	Training and support Governance Interoperability Data sharing and accurate patient baseline information Enhanced connectivity for all	Technology developers, system developers, commissioners, integrated care systems, and digital health boards
Value proposition	Complicated	The value to the patient and carer is likely to be significant, reducing burden and ensuring a sense of safety and security. Based on staff perspectives, while this high value to patients can be classified as simple, there is no evidence of desirability, safety, efficacy, or cost. The value to the system is currently uncertain, depending on many factors, including initial costs, cost-effectiveness, and impact on outcomes, safety, and efficacy. The value to clinicians is yet unknown, as no trial has been conducted in practice.	Proof-of-concept study Stakeholder engagement Patient and public involvement and engagement Qualitative study with patients and carers Case studies and evaluations around models of care, clinical effectiveness, patient outcomes, and time and economic implications, including environmental impact	Service leads, organizations, and commissioners
Adopters	Simple (workforce-perceived; patients/carers), complicated (staff)	As perceived by workforce participants, the DT would be more acceptable to patient and carer adopters who experience a lower burden. There are more factors to consider with staff adopters, as it would involve learning new skills and a change to routine tasks and potentially roles.	Education and training for patients, carers, and the workforce Embed training at the undergraduate level and placements for staff Stakeholder engagement Patient and public involvement and engagement DT champions Review and plan workflow, roles, and capacity	Service users, patients, carers, family members, and patient and public representatives (including patient and public involvement and engagement)
Organizations	Complex	In current health care organizations, systems are complex, with a varying appetite for innovation. This variance depends on many factors, including willingness for innovation, formal links across multiple organizations, shared vision, and budget implications. There is a need for well-led resources to establish successful adoption and readiness.	Education and training Linking between organizations Shared systems and interoperability Shared vision and pathways to avoid duplication of care Accessible funding and shared resources for the workforce and infrastructure 24-hour response pathways	Health system, organization board members, acute or community trusts, and integrated care systems
Wider system	Complex	Financial and regulatory requirements are not yet understood.	Governance including information governance, data security, cyber security, and ethical considerations Connectivity for the population	National representative bodies, HaH society, NHS England, and Department of Health and Social Care
Embedding and adaptation over time	Complicated	A positive outlook is present, with agreement that DTs align with national policy changes. Increased use of digital technology and adaptability will be seen while familiarity and an emerging evidence base develop. There will need to be robust resilience to deal with unforeseen circumstances and adaptations over time, as well as behavioral change.	Improved evidence base including proof of concept and evaluations Data management Internet connectivity Digital interoperability Raise shared awareness across an integrated care system Aligning with national and local strategies Collective action and reflective action	Clinicians, practitioners, integrated care systems, service users, and policymakers

a NASSS: Non-adoption, Abandonment, Scale-up, Spread, and Sustainability.

b HaH: hospital-at-home.

c DT: digital twin.

## Discussion

This study aimed to explore health and care professionals’ perceptions of a DT-enabled HaH model of care and its perceived impact. Although this technology has not yet been demonstrated in practice, data from participants were used to explore a conceptual model, providing an in-depth understanding of acceptability, benefits, and challenges across multiple levels: individual (patients and staff adopters), meso (organizational), and macro (systemic). The use of the NASSS framework, which is based on a synthesis of multiple theories of technology implementation [21], enabled a rich understanding of multiple sociotechnical factors influencing the potential adoption of DT technology and its integration within existing HaH models of care. The findings showed that there are many uncertainties and multilevel factors that can potentially impact adoption. Some include the current conceptual nature of HaH DTs and the unknowns around interoperability and integration. Furthermore, the potential change to staff routines and the need to learn new skills may involve new structures within organizations to embed DTs, leading to initial apprehension among staff. However, in general, professionals’ views of the concept were positive, and they reported that embedding DTs in practice over time was manageable.

This study highlights key themes to guide recommendations for stakeholder consideration, supporting the implementation of HaH DTs and long-term sustainability. First, the results show that improvements are required from a system-wide perspective to enable successful adoption, scale-up, and spread. These may include digital infrastructure, connectivity, and interoperability with a shared vision centered around the patient. These can enable true person-centered care and holistic insights, and facilitate the predictive capabilities of DTs, if accurate data are inputted. This could facilitate raising awareness across multiple organizations, which can support long-term sustainability. Additionally, supportive infrastructure includes human resources and workforce capacity, which may require remodeling and new ways of working to deal with large amounts of data. There is potential for hybrid roles, working across boundaries, and a level of flexibility. While there is a scarcity of studies around the adoption of DT technology in health care, particularly in a patient’s home, this study advances new knowledge that contributes to and builds upon what is already known about the adoption of existing technologies such as telehealth and remote monitoring. However, this study was conducted in a systematic manner using a robust implementation framework and differs conceptually by considering the potential leverage that DTs offer, such as real-time simulation and predictive analytics. Findings from studies examining telehealth adoption have noted that system-wide improvements and service enhancement or redesign are needed to achieve sustainability [31]. A study by Thomas et al [32], which explored telehealth adoption in Australia using the NASSS framework, similarly found that implementation strategies are needed at multiple levels to support this sociotechnical complexity. The authors noted the importance of raising awareness, as well as clinicians’ desires for flexible hybrid models, dedicated spaces, and structured staff training. The findings broadly correlate with the work of Sugarhood et al [33], who described that innovative technology cannot be seen as “plug and play” but as a complex innovation requiring strengthened linkage between different parts of the system, with input and coordination between people and organizations. This may require extensive changes to systems, structures, and ways of working, focusing on optimizing human-system integration. The authors describe the technology itself as a “hard core” (the unchangeable features of the innovation itself) that requires a “soft periphery” (systems and services essential to full implementation) [33]. Comparably, this “soft periphery” requires “organizational innovativeness” and receptiveness among organizations and leaders, which can vary [33]. Similar to our study, other research has described that technological innovations face challenges such as competing priorities and organizational financial pressures [33].

Second, adapting working models and using advanced technology require robust and ongoing training and support for reassurance, sense-making, and enhancing human-system interaction. Technical support and in-house DT champions were highlighted by participants as potential key facilitators to improve adoptability and provide supportive reassurance for any initial reluctance in the workforce. This training needs to be incorporated into clinicians’ undergraduate and heuristic training to ensure that DTs are used to complement and enhance care and do not replace clinical judgment or lead to overreliance. This aligns with the findings of Sugarhood et al [33], who conducted a key informant interview study exploring telecare technologies as a system innovation. Their study identified that technological innovations require people to think and behave differently, recognizing the importance of staff training and workforce development in adoption. However, while initial successes were noted with designated digital “champions,” these trained staff were found to often move on to new roles, impacting sustainability. Cartledge et al [31] highlighted that continuous support and improvement were key themes impacting adoption, particularly focusing on staff adopters and organizational domains.

Third, this study showed that adoption and implementation require reassuring evidence, suggesting that initial proof-of-concept studies can help identify any initial technical challenges before wider implementation. Participants felt that this would also help clarify which patients would be suitable for DTs, shaping any selection criteria. The recommendations for initial implementation suggest that stakeholder engagement is important throughout, with the involvement of individuals from various levels to cover all domains of the NASSS framework. Some of these may include patients, members of the public, clinicians, allied health practitioners, service leads, commissioners, technology developers and suppliers, digital leads, organization board members, and national policymakers. This is consistent with broader literature on technological adoption, which suggests that trialability (opportunity to experiment before committing to adopt) and observability (visible benefits) are both facilitators for adoption [33]. In the literature, examples for telecare include having a “show flat” or demonstration area attached to a day center for older people [33], and this approach could be considered for DTs in the community [10]. From a different angle, other literature has mentioned that new technology should not just focus on proof-of-concept but rather use co-production from the early stages of design and development to identify how technologies might better fit in with users’ material surroundings, networks, and lifestyles [33]. This is critical as the needs of end users may differ from the needs and capacities of providers [33].

The findings from this study contribute to the growing international literature on DTs, specifically addressing the scarce research on HaH. National drivers advocate for increased use of technology, care closer to home, and strategies for prevention [11]. However, remote monitoring and the use of technology in HaH remain variable and uncertain [7]. Moreover, it has been argued that increased use of technology can support increased use of HaH [7]. Maximizing the potential of technology requires recognizing that technology can automate time-consuming tasks, such as data entry, and safely manage larger patient groups with the use of remote monitoring [7,34]. In their systematic review, Shi et al [35] recognized that current pressures on urgent and emergency services are encouraging innovative care models that use technology, representing a paradigm shift in acute health care delivery [35]. However, available evidence on high-intensity technologies, such as wearables for remote monitoring, remains scarce [8,35].

The findings of this study are consistent with international literature by Serrano et al [36], who similarly reported that most practitioners support the use of remote monitoring technology and perceived that this will become increasingly relevant in the future and advantageous for clinical practice. Serrano et al [36] described common benefits, including continuous monitoring of patients to provide prompt care, efficient communication, and visualization of health trends within a patient’s own environment, allowing personalized care. Furthermore, they reported that the way data are presented via wearables or devices can influence patient understanding and self-care decisions [36]. Some challenges, such as increased workload, data inaccuracy, and higher patient anxiety, were also noted [36]. However, other studies, such as one exploring workforce perceptions on the use of a pregnancy remote monitoring intervention by Lanssens et al [37], found no actual increase in workload when using the technology. Additionally, the systematic review by Serrano et al [36] identified practitioners’ concerns regarding privacy, which was not a key focus found in our data. While these studies are not entirely comparable to studies of HaH DTs, they explored key components of DTs, such as sensing technology (remote monitoring) for data collection, communication in some aspects (telecare), and visualization using dashboards, which are also used in clinical practice. Nonetheless, there remains a lack of literature specifically addressing HaH DTs, particularly regarding predictive analytics and simulation capabilities.

The application of this multilevel integrated framework highlights some of the contextual challenges within complex health care systems, showing that findings do not always fit separately within individual domains. For example, the theme around trust impacts staff and patient adopters as well as the technology domain and value proposition, and it is recognized that trust strengthens with a growing evidence base and time. This also applies to training, which overlaps across multiple domains and has financial impacts that affect the value proposition, organization, wider system, and embedding over time. These domains should not be taken in isolation, but they are useful as a guide to uncover some of the complexities that need to be considered before implementation, ultimately informing action planning and targeted efforts. This builds on previous literature, which highlights that health care systems can be complex, such as the systematic review by Greenhalgh et al [38] that explored the diffusion of innovations in health service organizations. This reinforces that multiple components can impact the diffusion, dissemination, implementation, and sustainability of innovations [38]. This needs to be recognized as an ongoing process [39]. Moreover, different clinical specialities may have varying priorities in a system, and these need to be further understood. This can be embedded into the design and development process, as has been demonstrated by Waddell et al [40], who integrated the NASSS framework into a well-known design process, the double diamond, for their human-centered intervention. This recognizes that stakeholder involvement and an interdisciplinary approach are important throughout the process. In practice, organizations and teams applying DTs in HaH services may wish to use the NASSS-Complexity Assessment Toolkit (NASSS-CAT) [41] to understand their own context-specific complexity across each of the 7 NASSS domains. This also supports stakeholder engagement involving questioning to understand suitability, feasibility, the capacity of organizations, and the readiness of adopters, including patients. Furthermore, the toolkit can be used as an ongoing tool at different points in time.

The use of a diverse sample with a range of participants supported the aims of this study. Participants had varying experiences, both within and outside of HaH and across multiple settings. The included participants had experience as digital leads and information officers, with some having responsibilities for commissioning decisions and others having no prior experience of high-level technology use and having only clinical experience. However, this study may have attracted interest from keen participants who may be viewed as potential early adopters of technology or those who have negative experiences of technology, introducing potential sampling bias. Furthermore, the inclusion of only participants based in the United Kingdom may limit the generalizability of the findings to international contexts. Ultimately, participants’ perceptions were based on theorization of a DT concept rather than first-hand experience of this technology, meaning that further work tailored to specific contexts would be required.

In conclusion, the findings of this qualitative study show that the adoption of innovations, such as HaH DTs, is shaped by multiple factors across several levels. While there is a perception of great value to patient and carer adopters, based on perceived reassurance and reduction in the burden with DTs compared to other technologies, there is a need for patient and public involvement or co-production to strengthen the findings. The use of a robust framework in this study allowed us to uncover some of the challenges with physical, cognitive, and organizational ergonomics that may be present with a HaH-DT concept and assess how stakeholders can build on this moving forward. The findings of this study highlight that system-level improvements and proof-of-concept evaluations support adoption, and digital champions support ongoing sustainability, awareness, and training. This work provides an early foundation for understanding likely adoption, and further research and implementation efforts should prioritize stakeholder involvement to develop clear, actionable plans.

## Supplementary material

10.2196/96645 Multimedia Appendix 1 Comparison between the theoretical framework of acceptability (TFA) and the Non-adoption, Abandonment, Scale-up, Spread, and Sustainability (NASSS) framework.

10.2196/96645 Multimedia Appendix 2 Interview vignette (slides) and topic guide.

10.2196/96645 Multimedia Appendix 3 Coding extracts: inductive and deductive (steps 2 and 3).

10.2196/96645 Multimedia Appendix 4 Table mapping themes to the Non-adoption, Abandonment, Scale-up, Spread, and Sustainability (NASSS) framework.

10.2196/96645 Multimedia Appendix 5 Theme refinement according to the Non-adoption, Abandonment, Scale-up, Spread, and Sustainability (NASSS) framework.

10.2196/96645 Multimedia Appendix 6 Themes with descriptions and complexity.

10.2196/96645 Checklist 1 COREQ checklist.

## Abbreviations

COREQ: Consolidated Criteria for Reporting Qualitative Research
DT: digital twin
HaH: hospital-at-home
ICB: integrated care board
NASSS: Non-adoption, Abandonment, Scale-up, Spread, and Sustainability
NHS: National Health Service
NIHR: National Institute for Health and Care Research
RPM: remote patient monitoring
TFA: theoretical framework of acceptability
