# Supplementary material for: Workforce Perceptions of a Conceptual Hospital-at-Home Digital Twin: Qualitative Study
Source: JMIR Hum Factors. 2026 Nov 5;13:e96645. doi: 10.2196/96645 (PMC13738007; doi:10.2196/96645)
Supplement: Supplementary file 2 [file humanfactors-v13-e96645-s001.docx]

Multimedia Appendix 1. Comparison between the theoretical framework of acceptability (TFA) and the Non-adoption, Abandonment, Scale-up, Spread, and Sustainability (NASSS) framework.

| **Framework** | **Primary-Focus** | **Level of**  **Analysis** | **Key Components** | **Contribution to Combined Study** |
| --- | --- | --- | --- | --- |
| TFA | Acceptability of healthcare interventions from the user’s perspective | Micro  (Individual/Small Group | Affective attitude, burden, ethicality, intervention coherence, opportunity costs, perceived effectiveness, self-efficacy | Provides rich, detailed data on user feelings, beliefs, and confidence in the intervention |
| NASSS | Complexity of implementation, spread, and sustainability of technology-supported interventions | Meso/Macro  (Organisational/System) | Seven domains: condition, technology, value proposition, adopter system, organisation, wider system, and embedding over time | Offers a structured lens to assess organisational readiness, scalability, and sustainability of innovations |
