# Supplementary material for: Workforce Perceptions of a Conceptual Hospital-at-Home Digital Twin: Qualitative Study
Source: JMIR Hum Factors. 2026 Nov 5;13:e96645. doi: 10.2196/96645 (PMC13738007; doi:10.2196/96645)
Supplement: Supplementary file 3 [file humanfactors-v13-e96645-s002.docx]

| Slide 1 |  |  |
| --- | --- | --- |
| Slide 2 |  | - Describe patient (Aunt Susan, own bed/tv, own mug, cat) - Multidisciplinary team - A home visit - Technology like video calls, apps, wearables and other medical devices. |
| Slide 3 |  |  |
| Slide 4 |  | One Solution = DT  Not talking about clones  What is DT? – virtual replica of a system/object/ setting. Uses RT info and ML to make risk-predictions, feedback / visualize.  What do we need to know to create a DT – what is the key architecture? |
| Slide 5 |  | Some examples in current H/C DT  Radiotherapy for oropharyngeal carcinoma  Cardiac – TAVR simulating which aortic valve prosthesis |
| Slide 6 |  |  |
| Slide 7 |  | Allow efficient prediction, detection and guiding care management decisions |

**Semi-Structured Interview Guide- Perceptions of HCPs on H@H DT**

**Pre-interview housekeeping**

**Check:**

- Consent forms received, completed and that PIS was received
- Facilities for recording are suitable

**At the interview**

**Prior to recording- Introduction**

- Interviewer introduces themselves to participant prior to recording
- Thank participant
- Check participant has received PIS and check still gives consent to participate (verbal)
- Re-iterate aims of the study
- Confirm we would like to audio-record the study
- Emphasise anonymity
- Offer the opportunity to ask questions
- Interviewer to present a background to Hospital at home and potential of Digital Twin Technology.

**Start Recording - notify participant**

1 **Themes for Interview topics**

**1. Perceptions of the H@H Digital Twin technology**

Prompts:

- What are your initial thoughts towards DT technology?
- In your current practice- What would you say is your level of experience with digital solutions?

**2. Perceived benefits and challenges of using DT technology in Hospital at Home.**

Prompts:

- What do you think are the potential opportunities of DTs supporting care pathways or care decisions?
- Do you think they will be sustainable in health services if adopted?

**3. The acceptability of a H@H Digital twin concept amongst healthcare professionals**

Prompts:

- How do you feel about a H@H DT?
- What are your thoughts on their likely effectiveness?
- How do you think they will impact on staff or patient burden?
- Do you have any thoughts on what ethical considerations are needed for something like this in practice?
- Does the H@H DT fit well within current models or what needs to be understood or changed? (Intervention coherence)
- What are the perceived benefits and values that DT may bring in H@H models?
- Do you think you or your team will be willing to adopt DT technology in H@H?

**4. Impact of H@H DT on care decision-making, patient care flows in H@H pathways and workload capacity.**

- How do you think this technology will influence decision-making, support patient flow?
- What is the potential impact on clinical responsibilities and workload?

**5. How technology may impact patient outcomes, quality of care and safety.**

- What impact do you think DT will have on personalisation and patient-centred care and quality of care?
- What impact do you think DT will have on care continuity?
- What impact do you think DT will have on patient outcomes?
- What impact do you think DT will have on patient safety?

**6. Barriers and facilitators of adoption and integration within existing H@H models in the care system.**

Prompts:

- Do you have any concerns about data management, governance, interoperability?
- What training and resources do you think are needed?

**Post-interview housekeeping**

- At the end of the interview- notify when the audio-recorder is switched off and stop recording
- Thank participant for time and contribution
- Offer the opportunity to ask questions and to be kept informed of study progress**.**
