# Supplementary material for: Workforce Perceptions of a Conceptual Hospital-at-Home Digital Twin: Qualitative Study
Source: JMIR Hum Factors. 2026 Nov 5;13:e96645. doi: 10.2196/96645 (PMC13738007; doi:10.2196/96645)
Supplement: Supplementary file 4 [file humanfactors-v13-e96645-s003.docx]

Multimedia Appendix 3

Figure S1. Extract from early-stage inductive coding in NVIVO (Step 2)

Figure S2. Grouping codes to generate initial themes from the data (Stage 3)

Figure S3. Deductively applying the NASSS framework (Step 3)
