# Supplementary material for: Workforce Perceptions of a Conceptual Hospital-at-Home Digital Twin: Qualitative Study
Source: JMIR Hum Factors. 2026 Nov 5;13:e96645. doi: 10.2196/96645 (PMC13738007; doi:10.2196/96645)
Supplement: Supplementary file 5 [file humanfactors-v13-e96645-s006.docx]

Appendix 6. Themes integrated to NASSS Framework domains, level of complexity and descriptions

| **NASSS Category**  **(and complexity level)** | **NASSS**  **sub-category** | **Analytical Themes** | **Description of Analytical Theme** |
| --- | --- | --- | --- |
| **1. CONDITION**  **(Complex)** |  | **Right patients, Right problems** | Participants had mixed thoughts on who DT might be best for. Maybe a H@H-DT is not right for those with cognitive or learning disabilities. Some say multimorbidity would pose too much complexity, whereas others say that the complexity may be better understood with the use of data input and outputs of DTs. |
|  |  | **Patient education and self-empowerment** | Educating patients about their condition empowers them for better self-management |
|  |  | **Timely identification of risk and deterioration** | DTs help clinicians detect a patients' decline and how to deal with this. Useful for risk identification or risk triage |
| **2. TECHNOLOGY**  **(Complex)** |  | **Access and Connectivity** | Availability of WiFi access and connectivity is crucial to support DTs to all members of the population equally. Also a willingness to engage with internet and the implications of patient financial conditions and signal interruptions can be foreseen challenges, which should be equitably resolved. |
|  |  | **Trusting Device Accuracy** | Trust in the device and the data it uses/ generates as an accurate and credible source to support clinical- decision-making and safe practice. Impact on clinical accountability. Ensuring that the technology and any AI is trained with accurate information (coding accuracy of medical data). Ensuring patient variability is considered. Reliability also needs ongoing maintenance of devices (e.g. calibration) |
|  |  | **System alerts and frequency of monitoring** | Alerts generated by the DT system need to be accurate and helpful. Many participants highlighted that continuous monitoring may be un-necessary and that timed monitoring and triggered interventions are more practical. |
|  |  | **Training to support readiness and informed decisions** | Training is crucial for the workforce and the public on DT technology to support digital readiness. Patients should also have an informed choice, and other avenues should remain open to support acceptability. |
| **3. VALUE PROPOSITION**  **(Complicated)** | **3.1. Both System & Individual** | **Evidence base to support use** | Participants felt that strong evidence-base justifying the positive benefits is needed as well as lived experiences of the workforce to support adoption. |
|  |  | **Improved access and equity of care due to personalisation** | Participants felt that DTs can facilitate equity of care and improve access due to personalisation and tailoring to patients within their context. For example, allowing care to be given to those 'hard to reach' or using AI and technology to communicate with those who have language barriers, etc. |
|  | **3.2 Patient and Carer** | **Real-time and connected data allowing personalisation and prevention** | This theme highlights DTs facilitating the right care at right time. Allowing preventative care, getting a holistic picture of the patient and using real-time data as a way of ‘connecting the dots' in the system to allow personalisation of care. Data-driven interventions. |
|  |  | **Automated tasks relieving patient and carer burden** | Participants felt that automated tasks and monitoring reduces burden on patients and families. This provides convenience and reassurance, and becomes empowering for patients if they can see how it benefits them. |
|  | **3.3. System** | **Support clinical decision-making and complement care** | Participants emphasised the importance of DTs being used as a decision-aid rather than replace clinicians and should embed education and training. It allows clear visualisation of the clinical condition, trajectory and supports patient conversations. |
|  |  | **Cost implications and longer-term benefits** | Participants had mixed views. The cost implications of the technology, the maintenance and monitoring, as well as wider cost implications (indirect costs), such as electricity bills and the environmental costs. However, others could see that initial investment is needed to regain the long-term benefit and return on investment.  The long-term strategical gains on the system were seen as potentially (1) forecasting in organisational workflows helps organisations plan resources better and (2) Population health data means better health management, targeted care, strategic planning, and long-term cost benefits to the wider healthcare system. |
| **4. ADOPTERS**  **(Staff- Complicated)**  **(Patients and Carers- Simple)** | **4.1. Staff** | **Trust, clinical accountability dependent on experience and digital readiness** | Participants had differences of opinion and felt that acceptability of DTs is variable dependent on confidence, trust, level of experience and digital literacy and readiness. Participants felt that with time, training and exposure to technology and AI, the current younger generations and upcoming generations will be more acceptable of DTs. Some participants had concerns that the practitioner's clinical instinct and judgement may be compromised, however they could see the benefits if they had the appropriate training and the technology was reliable.  Participants also highlighted the importance of staff DT advocates- ‘DT champions' to support adoptability and reduce the training burden on the workforce and organisation. |
|  |  | **Workforce adaptability and re-modelling** | Although some participants felt there may be greater influx of data that can impact workload and capacity, in some cases, even duplicating work, there was acknowledgment that the role of the practitioners will change. Therefore re-modelling and adaptability of staff needs to occur to make efficient use of resources, and saw perceived opportunities for remote working or hybrid jobs, moving some admin duties to more clinical duties, potentially relieving staff burden and NHS pressures. There was perceived potential for filling in gaps for human error and allowing a minimum standard of practice. There was also a perceived need for ongoing training and potential to learn a lot from the data about a patients normal variability. This allows thinking outside of normal clinical parameters, using social cues as measures of deterioration, e.g. step count, alteration in daily activities, reduced use of the stairs, going to the bathroom more often etc. |
|  |  | **Individualising Interfaces for staff (accessibility)** | Supporting staff by personalising interfaces for practitioners allowing them to visualise patient clinical outcomes (projected), dashboard needs to be tailored to staff - i.e. visually pleasing, colours or numbers, be able to vary |
|  | **4.2. Patients** | **Phases of acceptance with patients/carers over time** | Future generations being more accepting as they have grown up through a more technological age and the resistance/distrust of the current population. It has been acknowledged that there are phases in acceptance. |
|  |  | **Increasing anxiety/ prompting health-seeking behaviour** | Potential to increase anxiety and worry to patients, the worried well or those more health conscious- potentially increasing health-seeking behaviour. Managing patient expectations for those expecting a quick response. Reducing patient -clinician physical contact was perceived as potentially as patients feeling they are being denied care or questioning are they being given the same level of care. However, this can also mean that the more appropriate patients are being seen with visits if needed. |
|  |  | **Patient compliance with the device** | Compliance with wearables, patients unplugging devices or going out of the house. |
|  | **4.3. Carers** | **Reassurance for carers** | Reassuring for care home staff or informal caregivers, i.e. those who don't live with patients, seen as a 'security blanket' or ' safety net'. |
| **5. ORGANISATION(s) (capacity, readiness, implementation)**  **(Complex)** |  | **Impact on workflows, care delivery and capacity** | Participants had concerns that current organisational structures may struggle with capacity or readiness to deal with large amounts of data coming from DTs and mechanisms in which to be able to action responses. This is especially crucial where a response is needed over a 24-hour provision of care. The concerns about opening the floodgates in an already struggling system were perceived as potentially creating more work, however in a more mature system that has had the necessary training and evidence base there was perceived potential to make better use of resources, subsequently reducing burden on organisations and increasing capacity in the system. This relies on appropriate training and use of DTs as a tool to support care delivery. |
|  |  | **Contingency plans and smart use of resources supporting 24-hour provisions of care** | Participants emphasised that there needs to be clear contingency plans in the event of failure and have systems for smart use of resources to allow 24-hour provisions of care. This means linking with other care services and widespread awareness of DTs in an integrated care system. |
|  |  | **Supporting infrastructure, Digital Interoperability and Human/ Organisational resources** | Participants expressed the need to have the supporting infrastructure. This could be digital infrastructure or human/organisational resources.  The digital infrastructure needs digital enablement with integrated digital systems, compatibility and data sharing across systems. Baseline information and data sharing were seen as crucial for success of DTs.  Human resources to support a 24 hour service provision would need dedicated resource to monitor the data coming into the hub and a dedicated role for this. Furthermore, working with other teams (i.e. physios, OTs, dieticians, etc) and raising awareness of services across the system was important, which would also support population health management and improve access to services. |
| **6. WIDER SYSTEM (political/regulatory/inter-organisational)**  **(Complex)** |  | **Governance needed at all stages** | The need for information governance, data security, cyber security, hacking considerations and ethical considerations is paramount across the whole DT concept. |
|  |  | **Risk of widening inequalities (digital/social) and potential for abusing the system** | There is a risk of creating barriers, widening inequalities, worsening digital exclusion. Also, a risk of a way of patients abusing the system (i.e. to falsify medical information, housing conditions to get better social housing). |
| **7. EMBEDDING AND ADAPTATION OVER TIME**  **(Complicated)** |  | **Mindset shift and adaptability required** | Participants generally expressed a positive outlook agreeing that DTs align with the 10-year plan and long-term healthcare strategies. There was a focus on the need to embrace change and that this workforce culture will change with time, knowledge and adaptability. |
|  |  | **Practical implementation requires proof of concept and evidence before widening implementation** | Participants had differing views on short-term and long-term implementation. Participants generally agreed that evidence is needed (or proof of concept) for clinicians to trust the technology. This could be trialled initially in conditions with clear pathways for management, adopting a phased approach initially. However, dealing with the challenges such as internet connectivity, digital interoperability, shared awareness in an integrated care system, and giving the necessary education will allow the subsequent roll-out to all patients, improve access to care and reduce inequalities. |
